# Publisher Correction: In silico discovery of representational relationships across visual cortex

**DOI:** 10.1038/s41562-025-02370-8

**Published:** 2025-11-18

**Authors:** Alessandro T. Gifford, Maya A. Jastrzębowska, Johannes J. D. Singer, Radoslaw M. Cichy

**Affiliations:** 1 Institute of Psychology, Freie Universität Berlin, Berlin, Germany; 2 Einstein Center for Neurosciences Berlin, Charité – Universitätsmedizin Berlin, Berlin, Germany; 3 Bernstein Center for Computational Neuroscience, Humboldt-Universität zu Berlin, Berlin, Germany; 4 Berlin School of Mind and Brain, Humboldt-Universität zu Berlin, Berlin, Germany

**Keywords:** Neural encoding, Perception, Visual system

Correction to: *Nature Human Behaviour* 10.1038/s41562-025-02252-z, published online 25 June 2025.

In the version of the article initially published, the vertical dashed lines were missing from the two right-hand side graphs in Fig. 3b and have now been reinstated. In the left-hand side graph in Fig. 8d, the data points were shifted down below the *x* axis and have now been amended, as seen in Fig. 1. These corrections have been made to the HTML and PDF versions of the article.

**Fig. 1:**
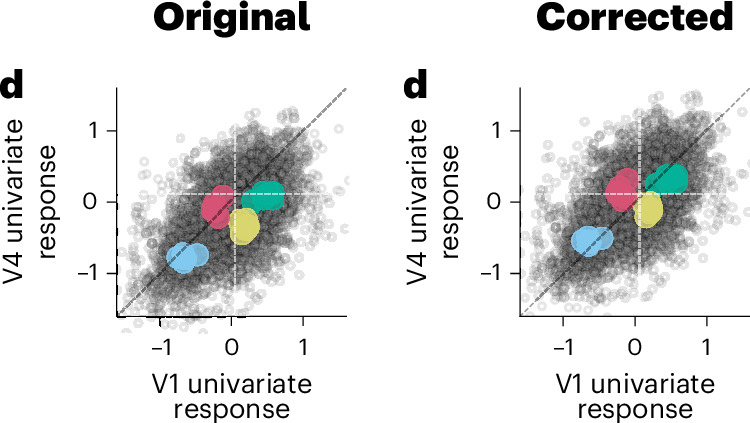
**Original and corrected Fig. 8d**.

