# Supplementary material for: In silico discovery of representational relationships across visual cortex
Source: Nat Hum Behav. 2025 Jun 25;9(10):2079–98. doi: 10.1038/s41562-025-02252-z (PMC12545174; doi:10.1038/s41562-025-02252-z)
Supplement: Supplementary file 1 — Supplementary Figs. 1–18 and Supplementary Tables 1–5. [file 41562_2025_2252_MOESM1_ESM.pdf]

nature human behaviour

Supplementary information

https://doi.org/10.1038/s41562-025-02252-z

In silico discovery of representational relationships across visual cortex

In the format provided by the authors and unedited

Supplementary Figure 1 | Results of univariate RNC applied on the in silico fMRI responses for the 50,000 ImageNet images, generated through encoding models trained on NSD.................... 2
Supplementary Figure 2 | Results of univariate RNC applied on the in silico fMRI responses for the 26,107 THINGS images, generated through encoding models trained on NSD...................... 3
Supplementary Figure 3 | Results of univariate RNC applied on the in silico fMRI responses for the 73,000 NSD images, generated through encoding models trained on the Visual Illusion Reconstruction dataset.. 4
Supplementary Figure 4 | Generative univariate RNC image solutions across generations, for one evolution.. 5
Supplementary Figure 5 | Generative univariate RNC without image complexity reduction.........6
Supplementary Figure 6 | Multivariante RNC optimization curves..7
Supplementary Figure 7 | Results of multivariate RNC applied on the in silico fMRI responses for the 50,000 ImageNet images, generated through encoding models trained on NSD............... 8
Supplementary Figure 8 | Results of multivariate RNC applied on the in silico fMRI responses for the 26,107 THINGS images, generated through encoding models trained on NSD................. 9
Supplementary Figure 9 | Results of multivariate RNC applied on the in silico fMRI responses for the 73,000 NSD images, generated through encoding models trained on the Visual Illusion Reconstruction dataset.. 10
Supplementary Figure 10 | Univariate RNC results for interactions between early-, mid-, and high-level visual areas.. 11
Supplementary Figure 11 | Univariate RNC results for interactions between early-, mid-, and high-level visual areas.. 12
Supplementary Figure 12 | Multivariate RNC results for interactions between early-, mid-, and high-level visual areas.. 13
Supplementary Figure 13 | Results of RNC applied on the in silico fMRI responses of high-level visual areas for the 50,000 ImageNet images, generated through encoding models trained on NSD.. 14
Supplementary Figure 14 | Results of RNC applied on the in silico fMRI responses of high-level visual areas for the 26,107 THINGS images, generated through encoding models trained on NSD 15
Supplementary Figure 15 | Multidimensional scaling (MDS) embeddings of the in silico univariate fMRI responses for controlling images found by applying univariate RNC on early- and mid-level visual areas.. 16
Supplementary Figure 16 | Multidimensional scaling (MDS) embeddings of the in silico univariate fMRI responses for controlling images found by applying univariate RNC on high-level visual areas.. 17
Supplementary Figure 17 | Multidimensional scaling (MDS) embeddings of the in silico multivariate fMRI responses for controlling images found by applying multivariate RNC on early- and mid-level visual areas.. 18
Supplementary Figure 18 | Multidimensional scaling (MDS) embeddings of the in silico multivariate fMRI responses for controlling images found by applying multivariate RNC on high-level visual areas.. 19
Supplementary Table 1 | Early and mid-level visual areas' retained voxels for in silico fMRI responses from encoding models trained on NSD.. 20
Supplementary Table 2 | High-level visual areas' retained voxels for in silico fMRI responses from encoding models trained on NSD...21
Supplementary Table 3 | Early and mid-level visual areas' retained voxels for in silico fMRI responses from encoding models trained on the Visual Illusion Reconstruction (VIR) dataset.... 22
Supplementary Table 4 | In vivo fMRI retained voxels for the univariate RNC experiment.........23
Supplementary Table 5 | In vivo fMRI retained voxels for the multivariate RNC experiment......24

Supplementary Figure 1 | Results of univariate RNC applied on the in silico fMRI responses for the 50,000 ImageNet images, generated through encoding models trained on NSD

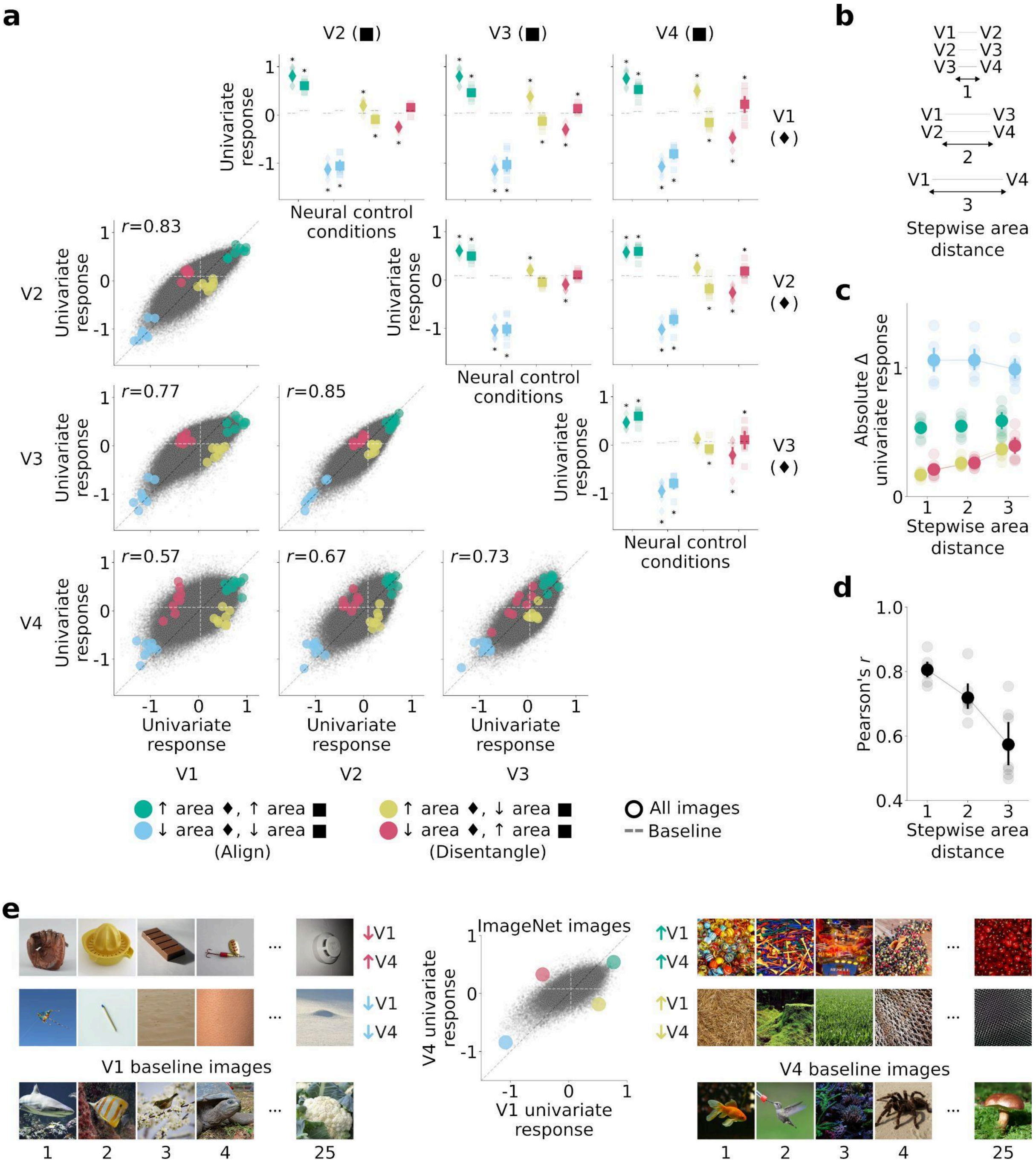

a, Univariate RNC quantitative results (univariate response magnitudes), embedded in a four-by-four matrix. **b**, Stepwise distance between areas. **c**, Absolute difference between controlling and baseline image univariate responses, averaged across all pairwise comparisons of areas with same stepwise distance. **d**, Correlation between the univariate responses of two areas, averaged across pairwise comparisons of areas with same stepwise distance. **e**, Univariate RNC controlling and baseline images for the V1 vs. V4 comparison. For copyright reasons, the original controlling images have been replaced with visually similar images from Pixabay.com and Pexels.com. The original controlling images are available on OpenNeuro (https://openneuro.org/datasets/ds005503).

Supplementary Figure 2 | Results of univariate RNC applied on the in silico fMRI responses for the 26,107 THINGS images, generated through encoding models trained on NSD

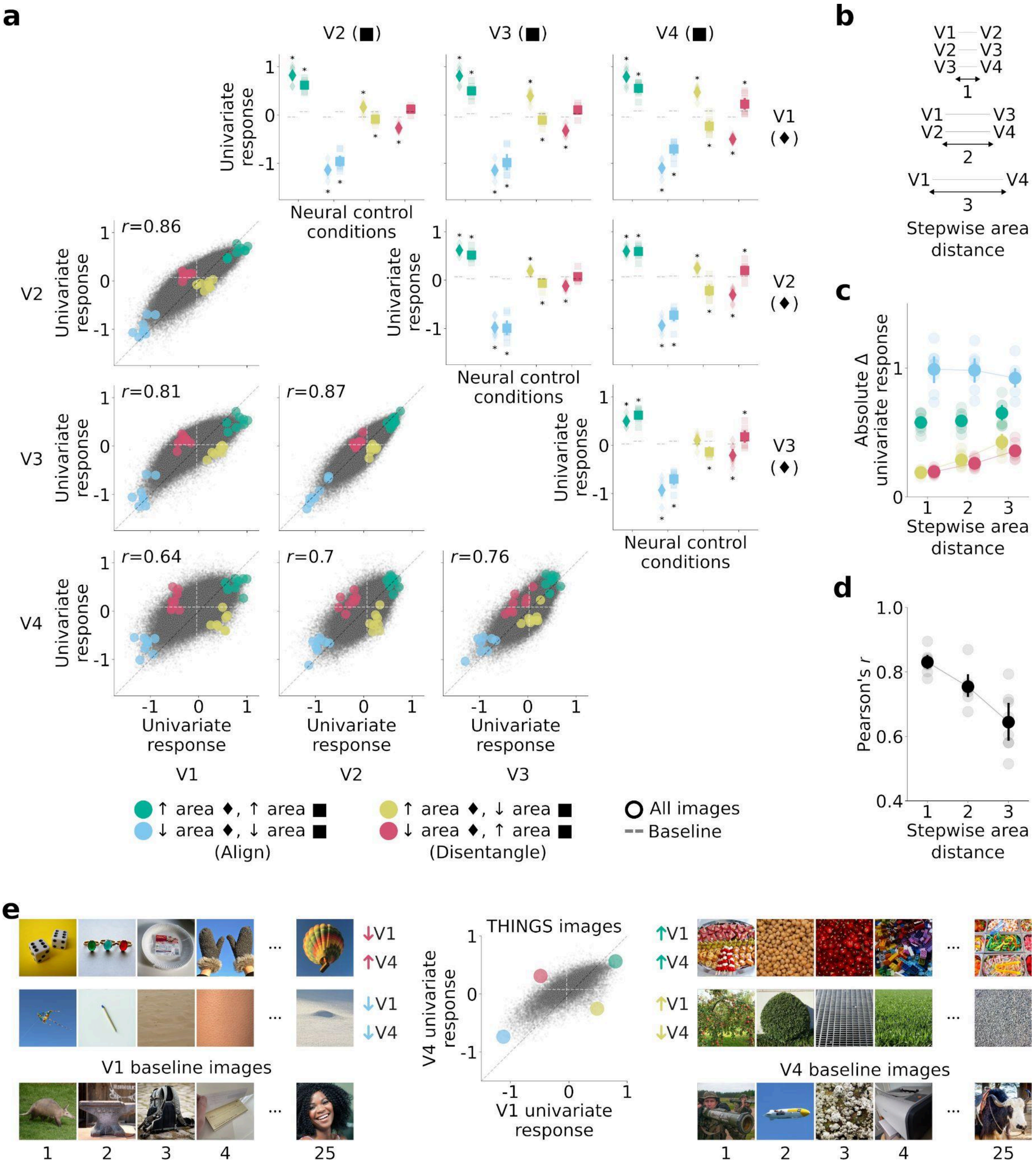

a, Univariate RNC quantitative results (univariate response magnitudes), embedded in a four-by-four matrix. **b**, Stepwise distance between areas. **c**, Absolute difference between controlling and baseline image univariate responses, averaged across all pairwise comparisons of areas with same stepwise distance. **d**, Correlation between the univariate responses of two areas, averaged across pairwise comparisons of areas with same stepwise distance. **e**, Univariate RNC controlling and baseline images for the V1 vs. V4 comparison. For copyright reasons, the original controlling images have been replaced with visually similar images from Pixabay.com and Pexels.com. The original controlling images are available on OpenNeuro (https://openneuro.org/datasets/ds005503).

Supplementary Figure 3 | Results of univariate RNC applied on the in silico fMRI responses for the 73,000 NSD images, generated through encoding models trained on the Visual Illusion Reconstruction dataset

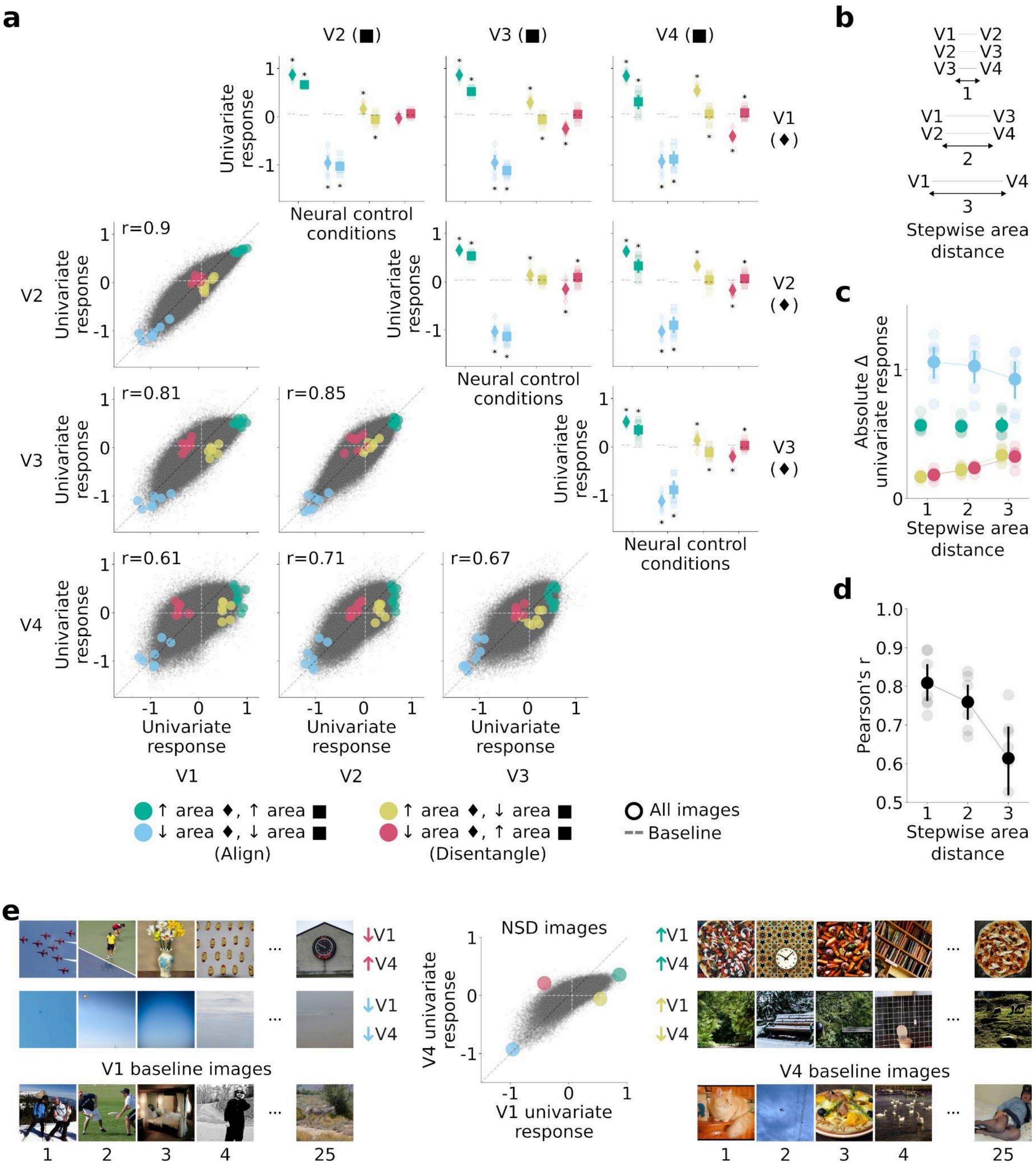

a, Univariate RNC quantitative results (univariate response magnitudes), embedded in a four-by-four matrix. **b**, Stepwise distance between areas. **c**, Absolute difference between controlling and baseline image univariate responses, averaged across all pairwise comparisons of areas with same stepwise distance. **d**, Correlation between the univariate responses of two areas, averaged across pairwise comparisons of areas with same stepwise distance. **e**, Univariate RNC controlling and baseline images for the V1 vs. V4 comparison.

Supplementary Figure 4 | Generative univariate RNC image solutions across generations, for one evolution

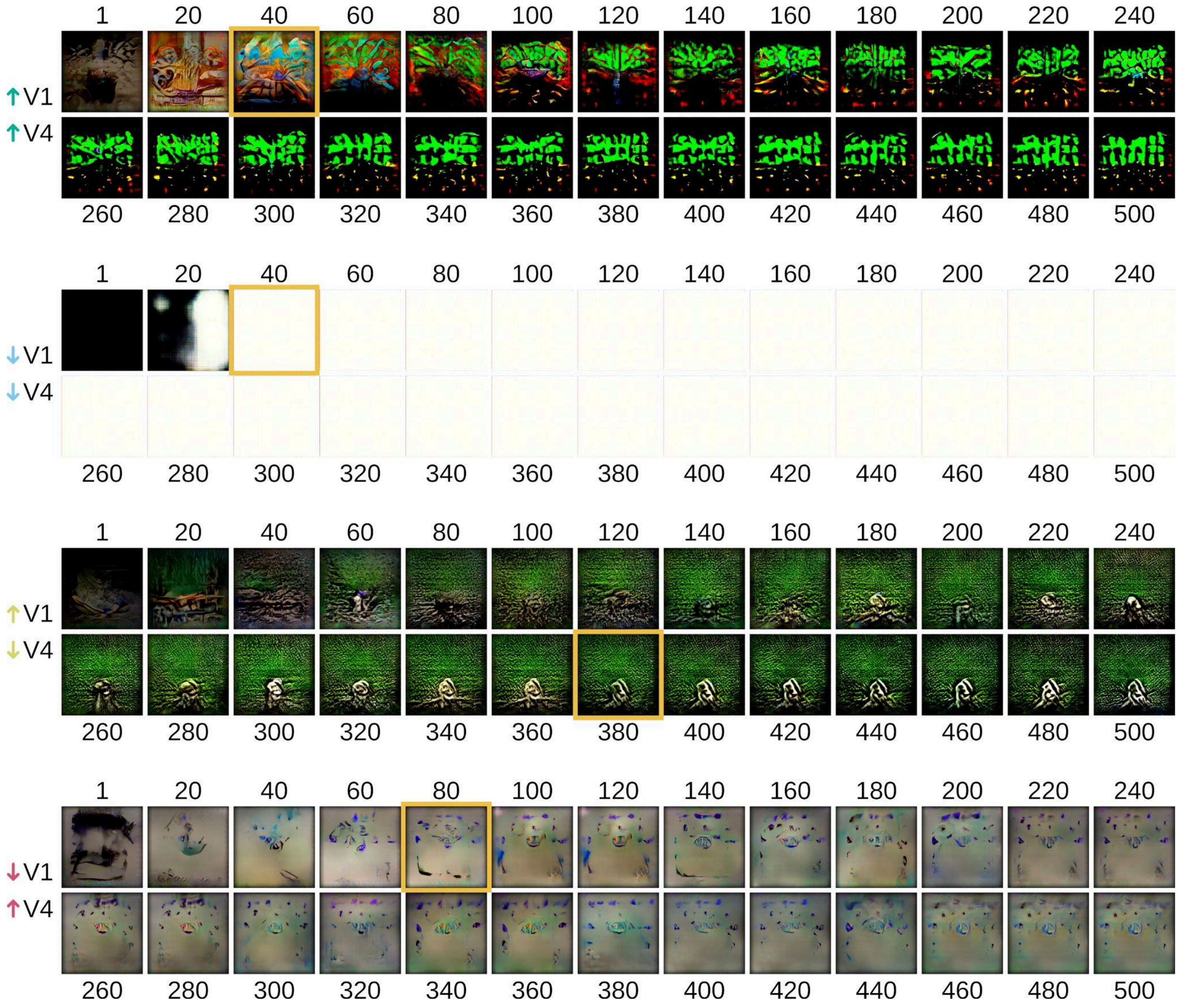

For each neural control condition, the generated images from the 500 genetic optimization generations are shown in intervals of 20 generations. Generation numbers are added above or below the images. Only the best performing image from each generation is displayed (out of all 1,000 images tested in each generation). The images are optimized to control univariate responses up to a threshold, after which they are optimized to reduce their PNG compression file sizes. The univariate response threshold is reached at generation 35 for the neural control condition driving both V1 and V4, at generation 32 for the neural control condition suppressing both V1 and V4, at generation 372 for the neural control condition driving V1 while suppressing V4, and at generation 76 for the neural control condition suppressing V1 while driving V4. For each neural control condition, the first post-threshold generation image is surrounded by a golden box.

Supplementary Figure 5 | Generative univariate RNC without image complexity reduction

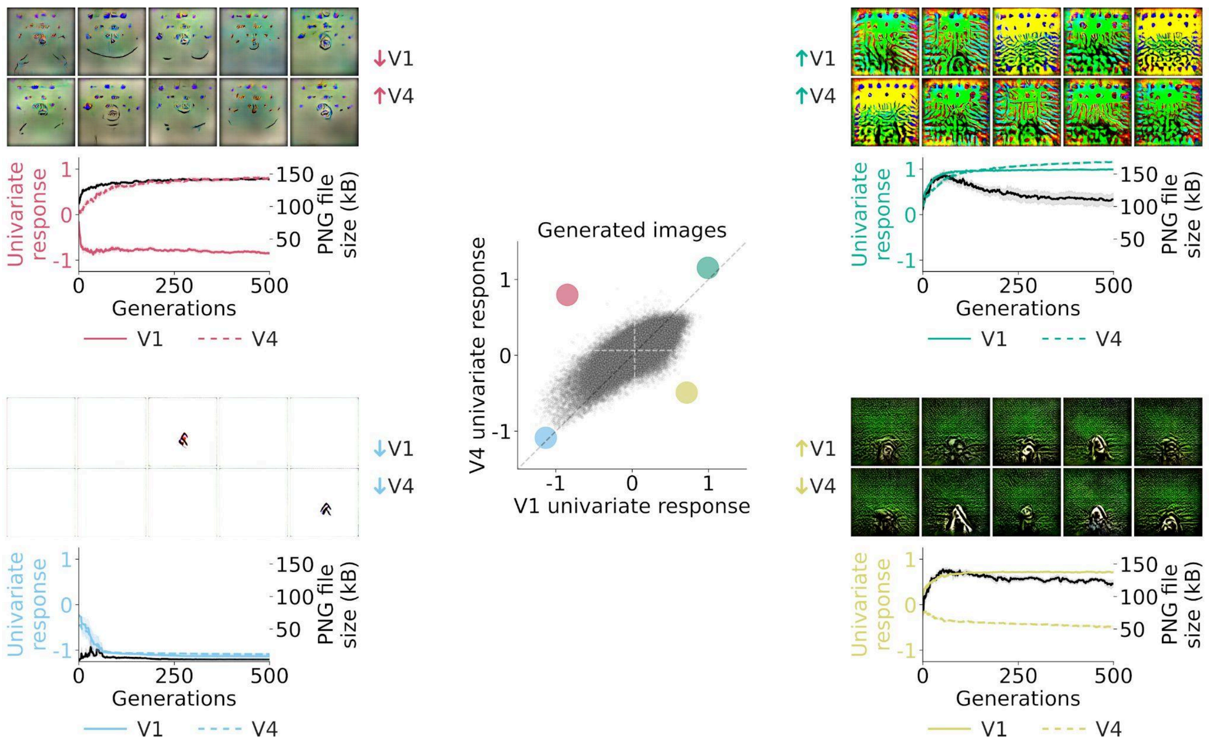

Results of ten independent generative univariate RNC evolutions using in silico fMRI responses averaged across all 8 participants. Across the 500 genetic algorithm generations, the images are only optimized to improve the neural control scores (i.e., the PNG compression file size is not reduced). For each neural control condition, the plots show the in silico univariate fMRI responses (represented by colored lines) and the PNG compression file size (represented by black lines) for the best GAN-generated image of each genetic algorithm generation, averaged across evolutions. On top of each plot are the optimized images from the ten evolutions.

Supplementary Figure 6 | Multivariante RNC optimization curves

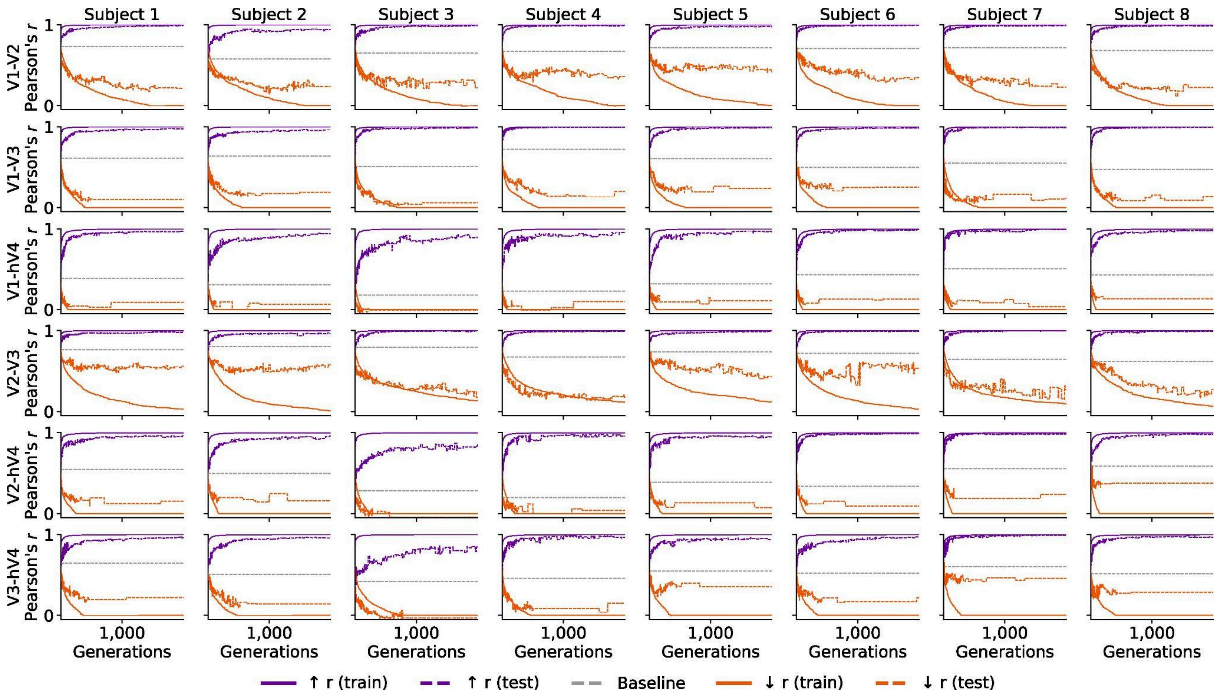

Optimization curves of multivariate RNC applied on the in silico fMRI responses for the 73,000 NSD images. Each row corresponds to a different pairwise comparison of areas, and each column to a different participant. The train curves indicate the neural control condition scores for the participant-average RSMs on which multivariate RNC was applied, and the test curves indicate the neural control condition scores for the remaining participant on which the multivariate RNC solutions were cross-validated.

Supplementary Figure 7 | Results of multivariate RNC applied on the in silico fMRI responses for the 50,000 ImageNet images, generated through encoding models trained on NSD

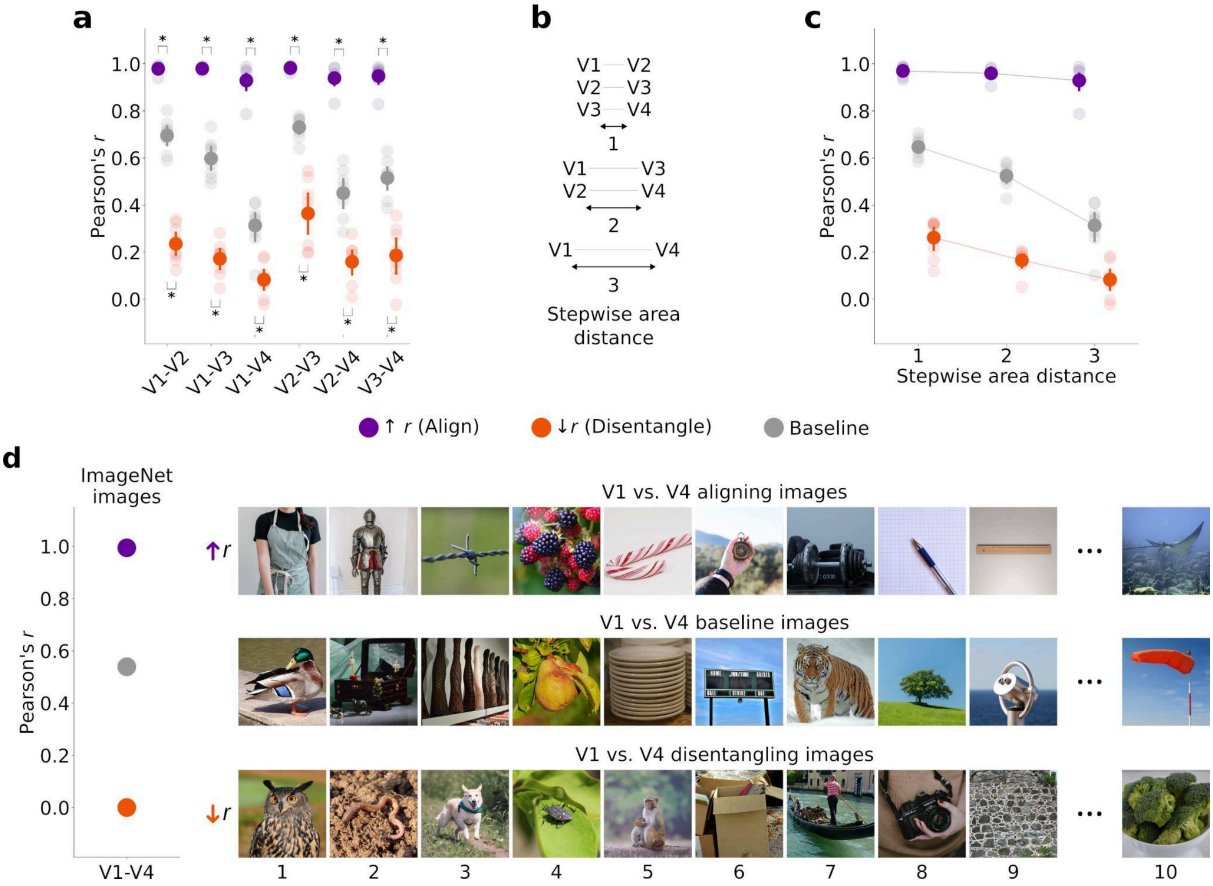

a, Multivariate RNC quantitative results (RSA scores). **b**, Stepwise distance between areas. **c**, Multivariate RNC RSA scores, averaged across pairwise comparisons of areas with same stepwise distance. **d**, Controlling and baseline images for the V1 vs. V4 comparison. For copyright reasons, the original controlling images have been replaced with visually similar images from Pixabay.com and Pexels.com. The original controlling images are available on OpenNeuro (https://openneuro.org/datasets/ds005503).

Supplementary Figure 8 | Results of multivariate RNC applied on the in silico fMRI responses for the 26,107 THINGS images, generated through encoding models trained on NSD

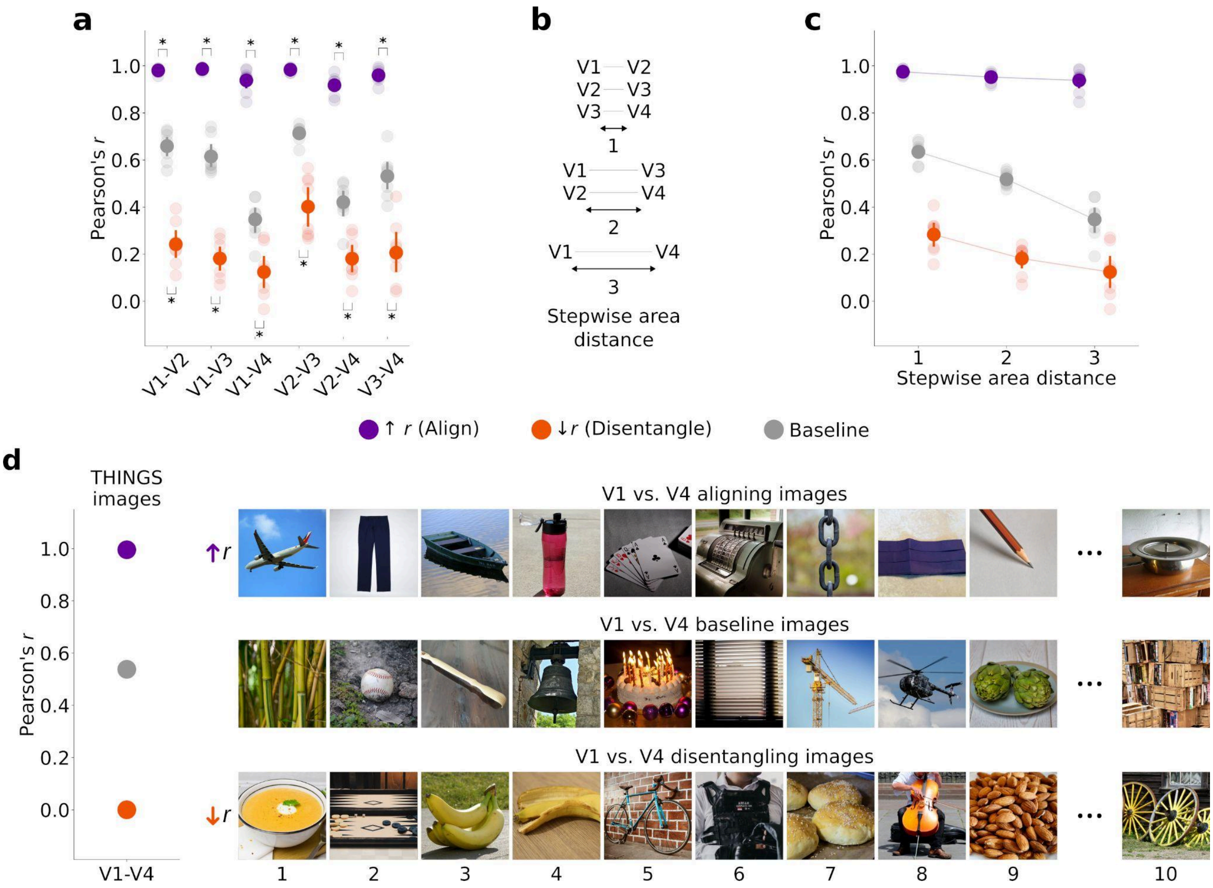

a, Multivariate RNC quantitative results (RSA scores). **b**, Stepwise distance between areas. **c**, Multivariate RNC RSA scores, averaged across pairwise comparisons of areas with same stepwise distance. **d**, Controlling and baseline images for the V1 vs. V4 comparison. For copyright reasons, the original controlling images have been replaced with visually similar images from Pixabay.com and Pexels.com. The original controlling images are available on OpenNeuro (https://openneuro.org/datasets/ds005503).

Supplementary Figure 9 | Results of multivariate RNC applied on the in silico fMRI responses for the 73,000 NSD images, generated through encoding models trained on the Visual Illusion Reconstruction dataset

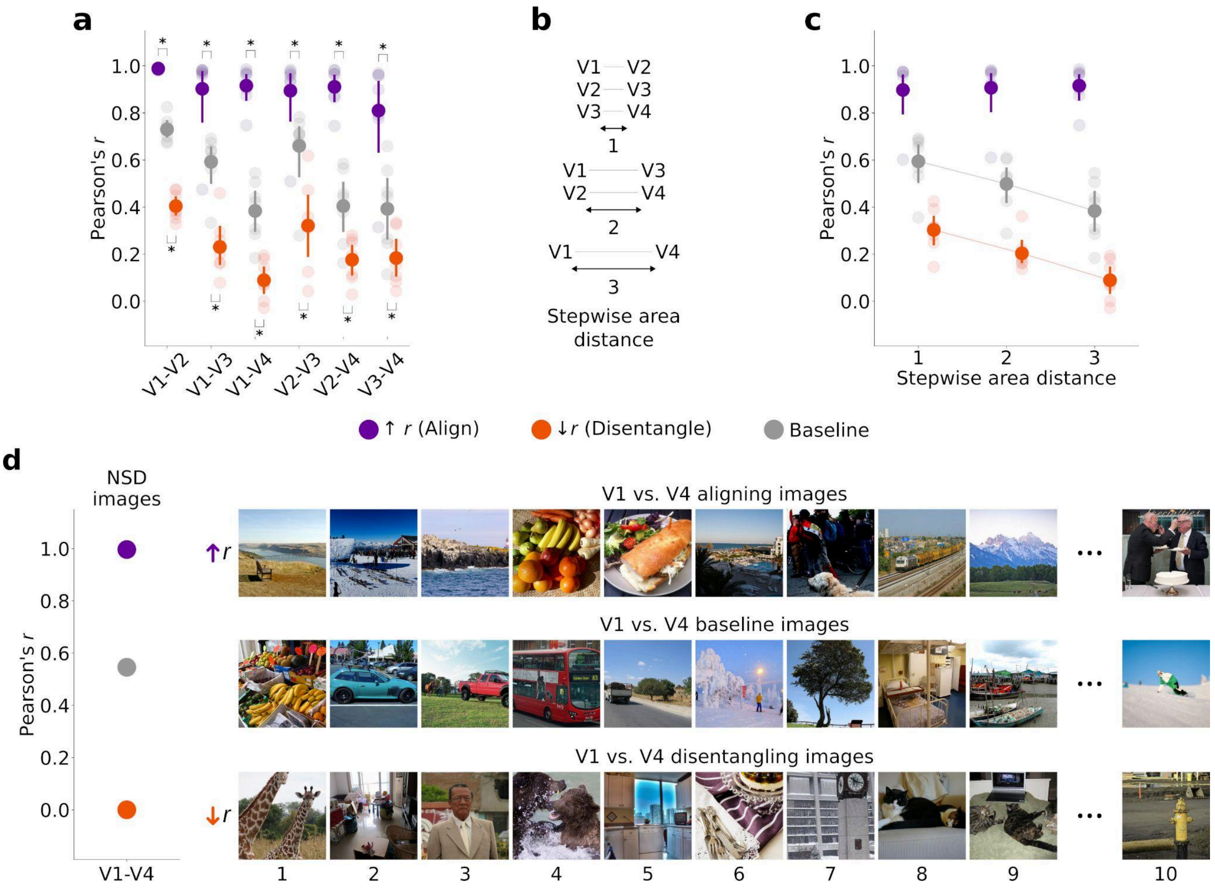

a, Multivariate RNC quantitative results (RSA scores). **b**, Stepwise distance between areas. **c**, Multivariate RNC RSA scores, averaged across pairwise comparisons of areas with same stepwise distance. **d**, Controlling and baseline images for the V1 vs. V4 comparison.

Supplementary Figure 10 | Univariate RNC results for interactions between early-, mid-, and high-level visual areas

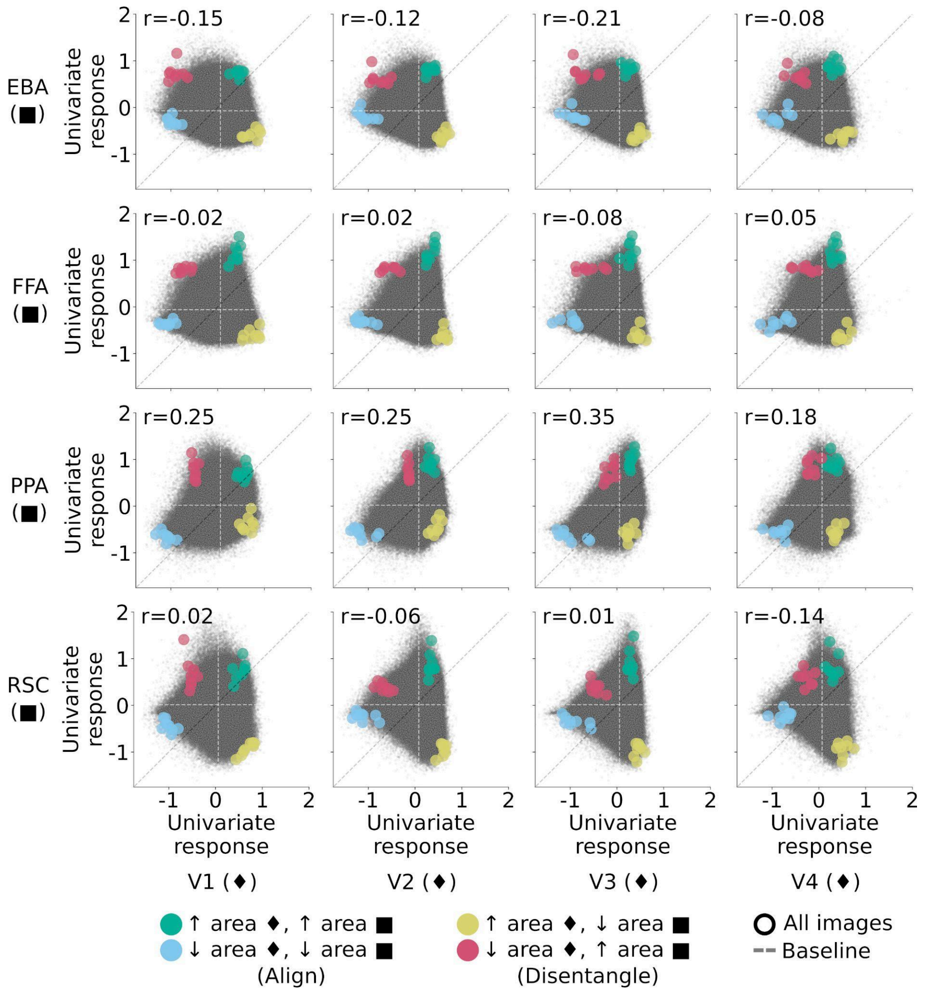

Univariate response image manifolds. Colored dots indicate in silico univariate fMRI responses averaged across the controlling images of each neural control condition, and small black points indicate in silico univariate fMRI responses of all participants for all 73,000 NSD images. Vertical and horizontal dashed lines indicate participant-average univariate response baseline for each area.

Supplementary Figure 11 | Univariate RNC results for interactions between early-, mid-, and high-level visual areas

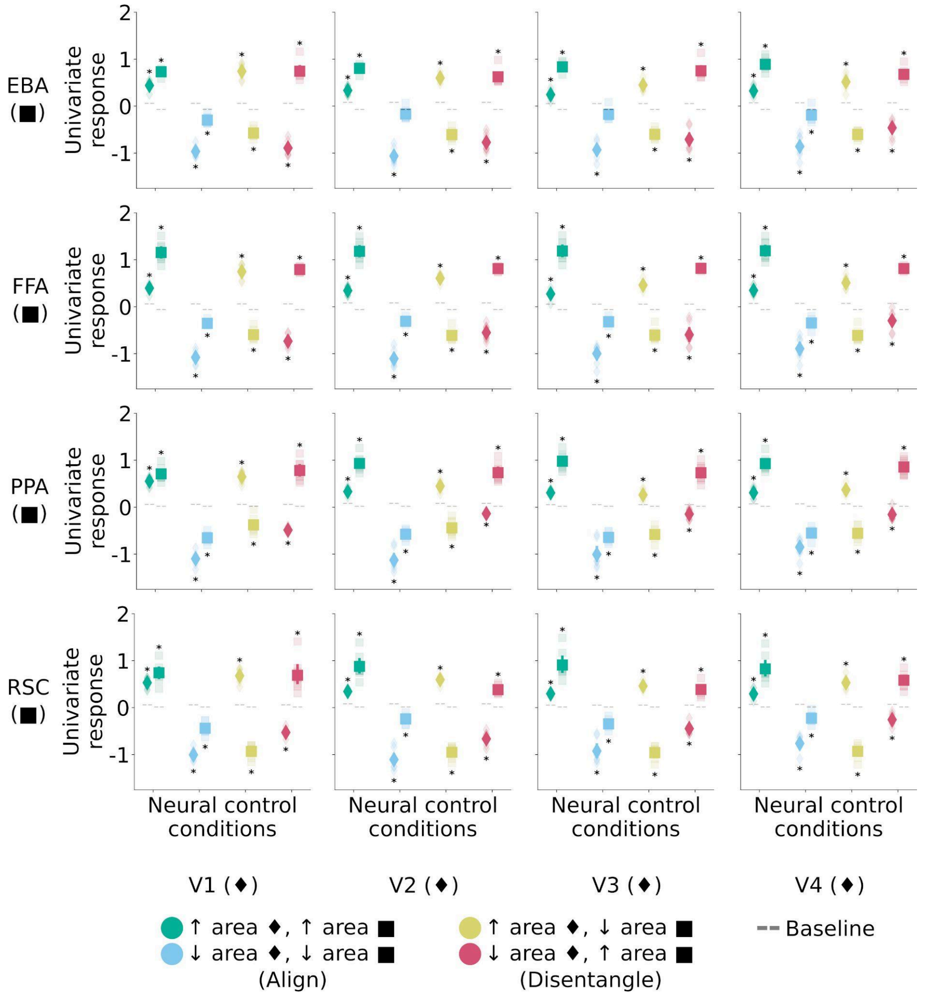

Univariate responses for the controlling images against the baseline. Diamonds and squares indicate the univariate responses of the areas indexed by the rows and columns of the results matrix, respectively.

Supplementary Figure 12 | Multivariate RNC results for interactions between early-, mid-, and high-level visual areas

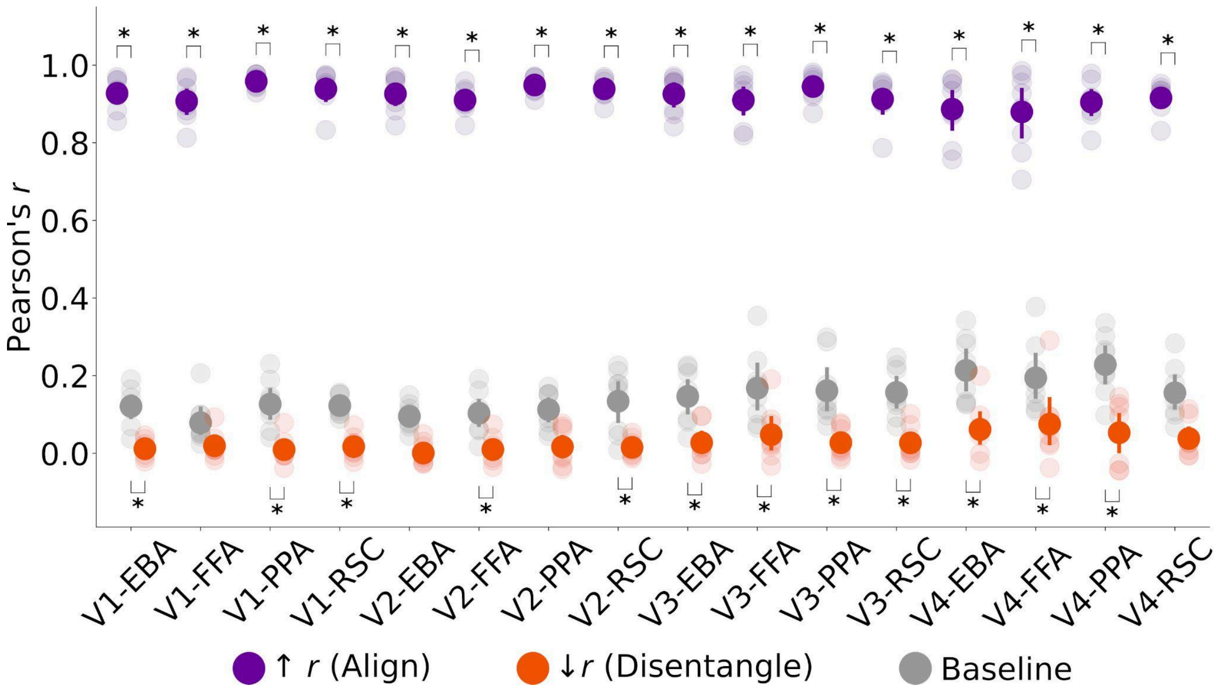

Multivariate RNC results, consisting of RSA scores (Pearson's *r*) for each pairwise comparison of areas.

Supplementary Figure 13 | Results of RNC applied on the in silico fMRI responses of high-level visual areas for the 50,000 ImageNet images, generated through encoding models trained on NSD

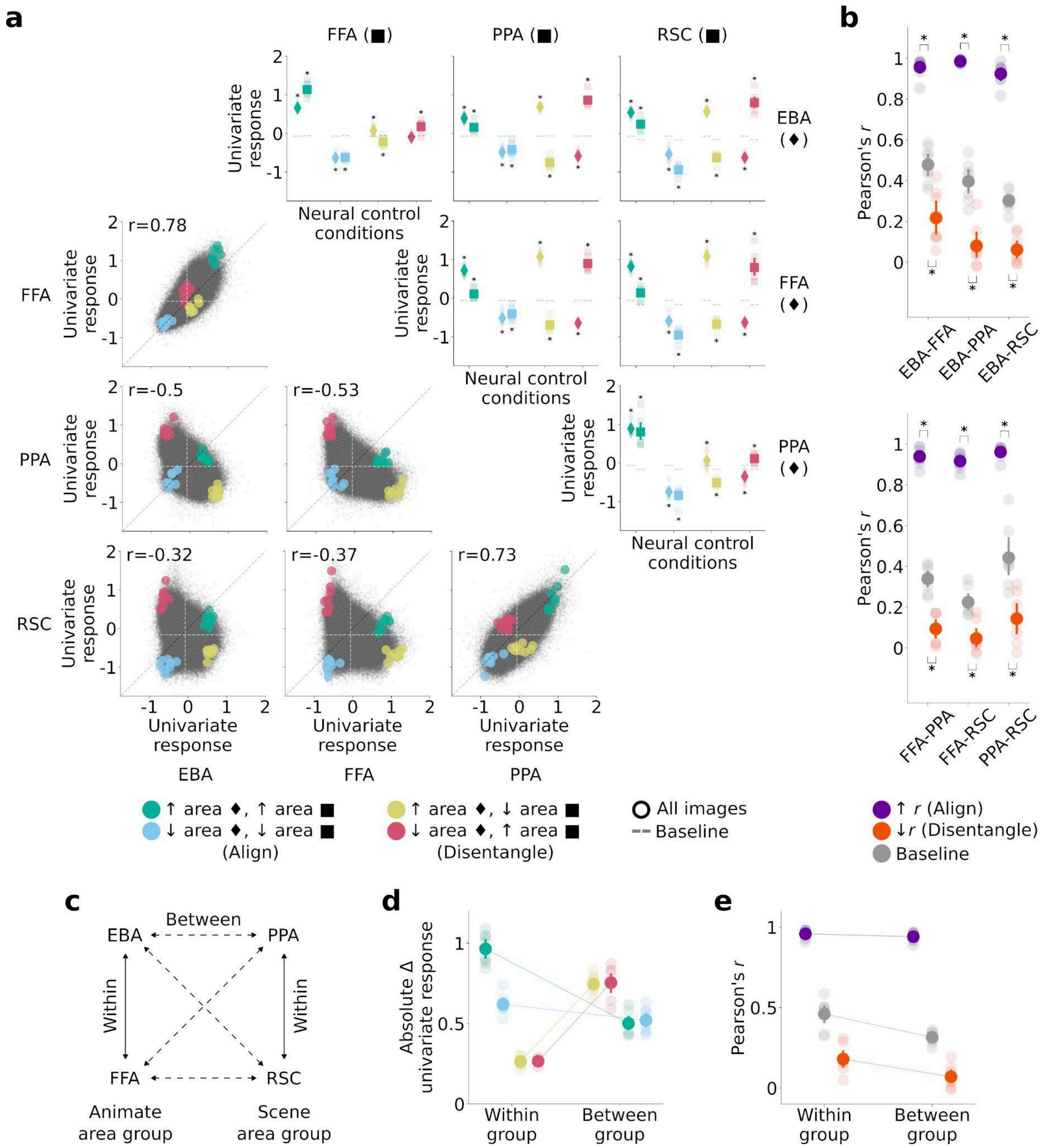

a, Univariate RNC quantitative results (univariate response magnitudes), embedded in a four-by-four matrix **b**, Multivariate RNC quantitative results (RSA scores) **c**, Categorical selectivity groups. Solid and dashed lines represent within- and between- group area comparisons, respectively. **d**, Absolute difference between controlling and baseline image univariate responses, averaged across within- or between-group area comparisons. **e**, Multivariate RNC RSA scores, averaged across within- or between-group area comparisons.

Supplementary Figure 14 | Results of RNC applied on the in silico fMRI responses of high-level visual areas for the 26,107 THINGS images, generated through encoding models trained on NSD

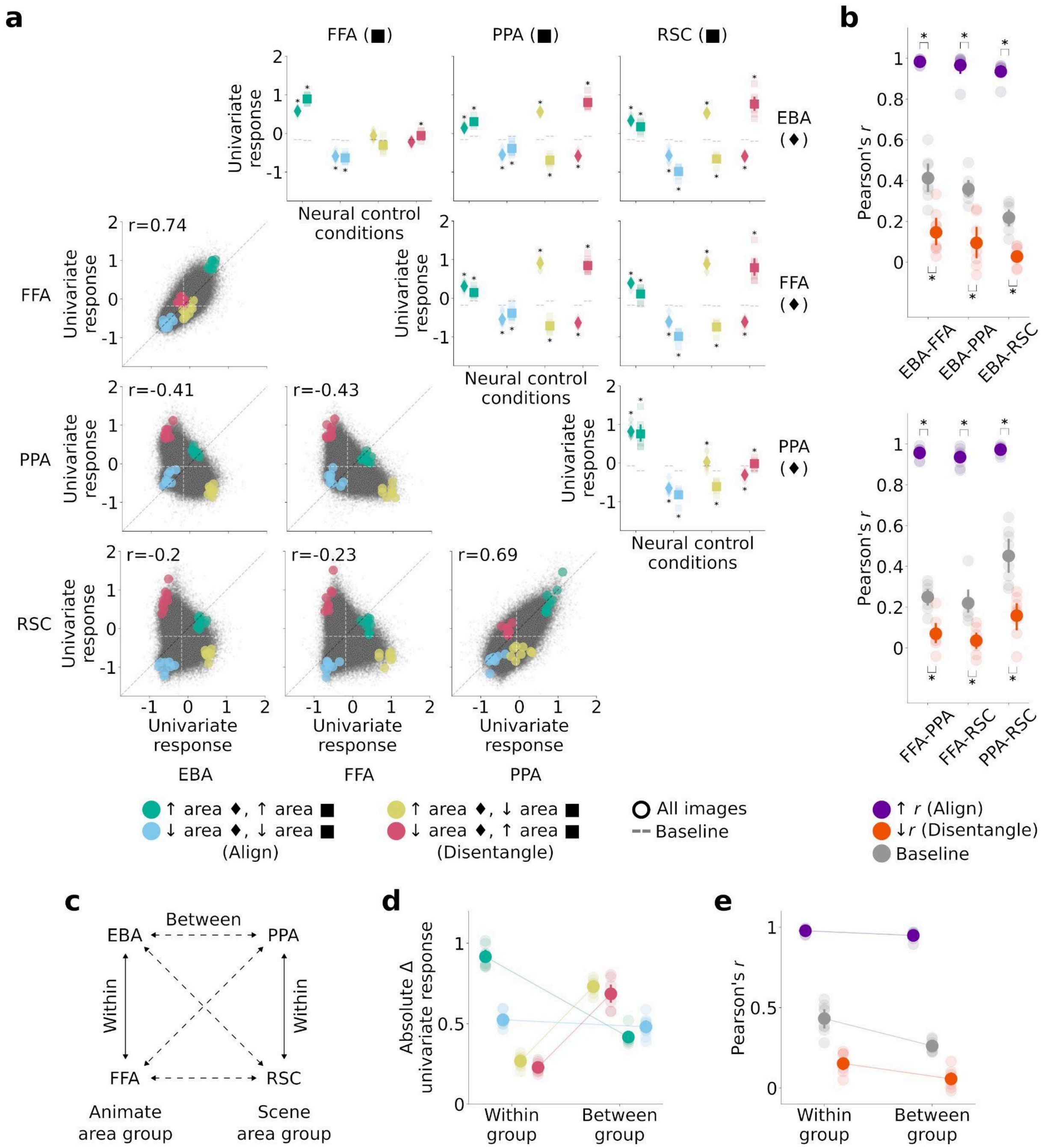

a, Univariate RNC quantitative results (univariate response magnitudes), embedded in a four-by-four matrix **b**, Multivariate RNC quantitative results (RSA scores) **c**, Categorical selectivity groups. Solid and dashed lines represent within- and between- group area comparisons, respectively. **d**, Absolute difference between controlling and baseline image univariate responses, averaged across within- or between-group area comparisons. **e**, Multivariate RNC RSA scores, averaged across within- or between-group area comparisons.

Supplementary Figure 15 | Multidimensional scaling (MDS) embeddings of the in silico univariate fMRI responses for controlling images found by applying univariate RNC on early- and mid-level visual areas

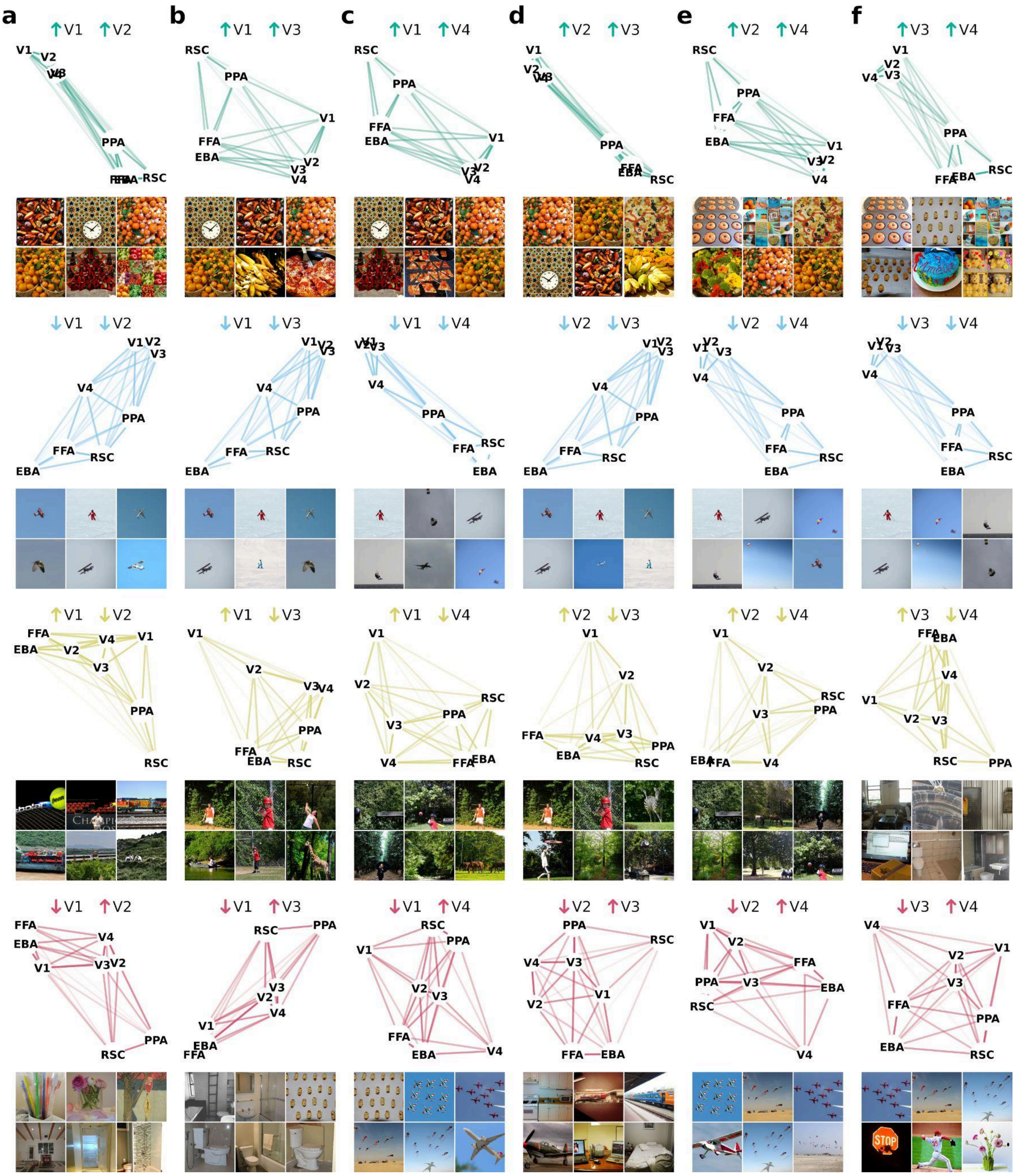

Six exemplar controlling images are shown for each control condition (all images come from the 73,000 NSD images). Each panel reflects MDS results and controlling images for a different pairwise comparison of areas. **a**, V1 vs. V2 comparison. **b**, V1 vs. V3 comparison. **c**, V1 vs. V4 comparison. **d**, V2 vs. V3 comparison. **e**, V2 vs. V4 comparison. **f**, V3 vs. V4 comparison.

Supplementary Figure 16 | Multidimensional scaling (MDS) embeddings of the in silico univariate fMRI responses for controlling images found by applying univariate RNC on high-level visual areas

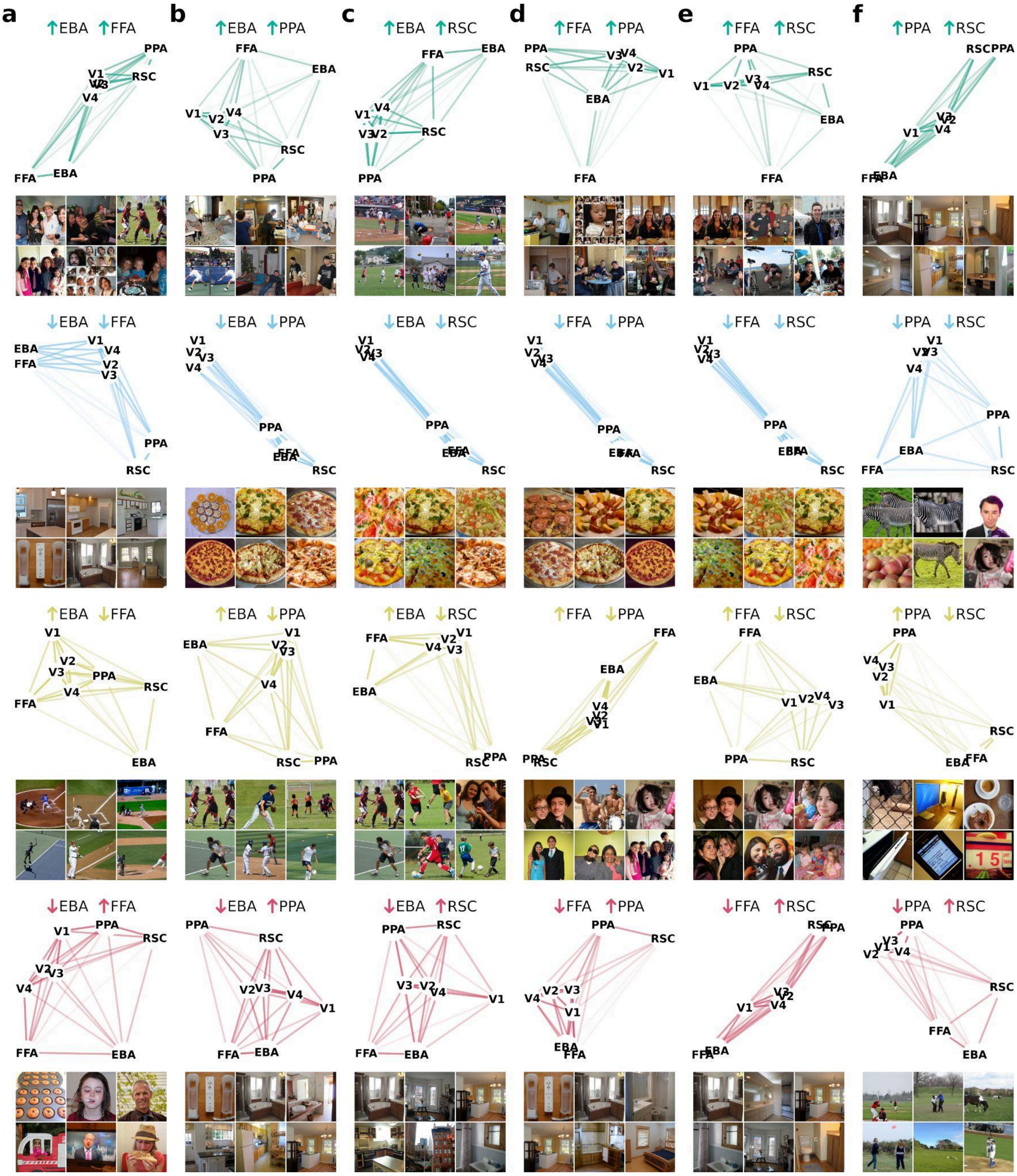

Six exemplar controlling images are shown for each control condition (all images come from the 73,000 NSD images). Each panel reflects MDS results and controlling images for a different pairwise comparison of areas. **a**, EBA vs. FFA comparison. **b**, EBA vs. PPA comparison. **c**, EBA vs. RSC comparison. **d**, FFA vs. PPA comparison. **e**, FFA vs. RSC comparison. **f**, PPA vs. RSC comparison.

Supplementary Figure 17 | Multidimensional scaling (MDS) embeddings of the in silico multivariate fMRI responses for controlling images found by applying multivariate RNC on early- and mid-level visual areas

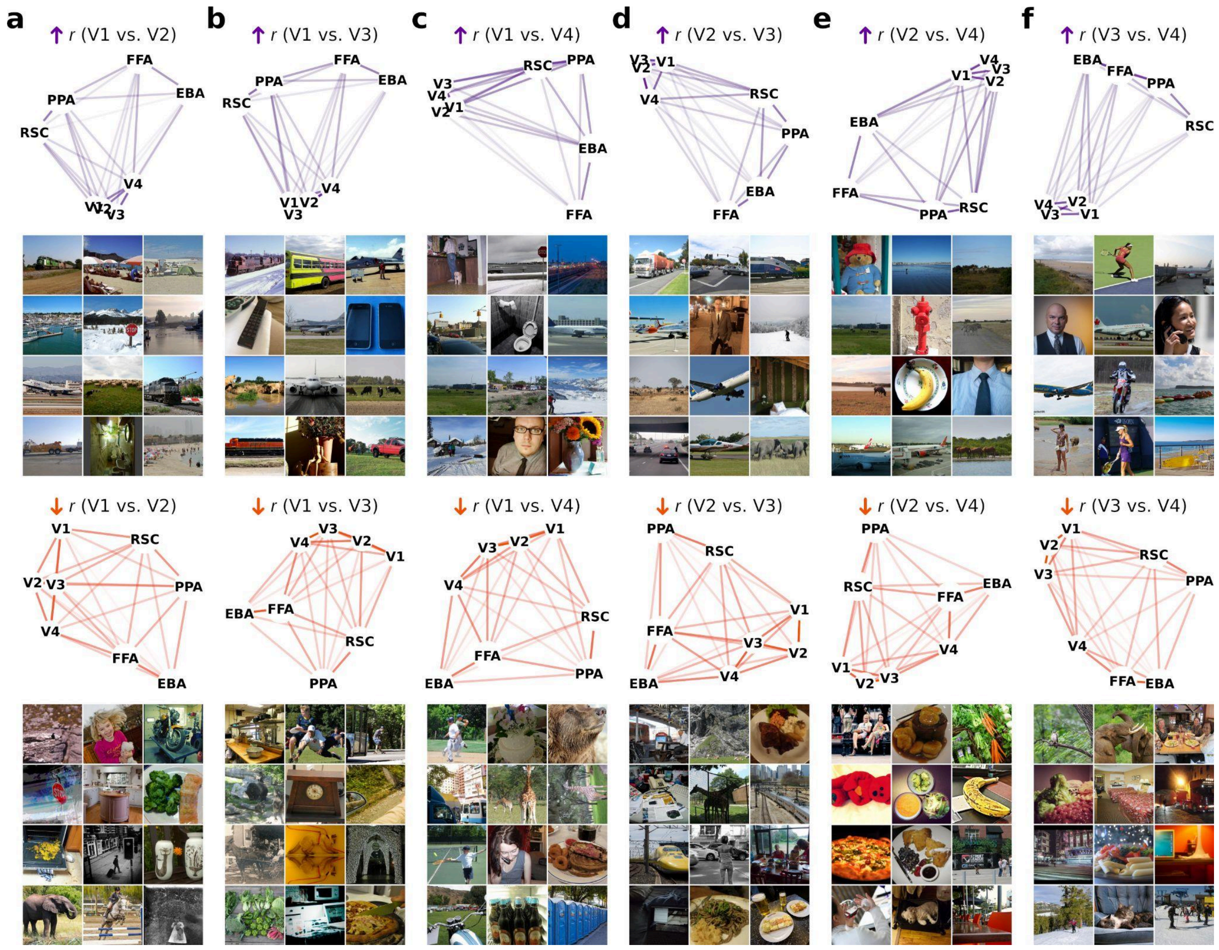

Twelve exemplar controlling images are shown for each control condition (all images come from the 73,000 NSD images). Each panel reflects MDS results and controlling images for a different pairwise comparison of areas. **a**, V1 vs. V2 comparison. **b**, V1 vs. V3 comparison. **c**, V1 vs. V4 comparison. **d**, V2 vs. V3 comparison. **e**, V2 vs. V4 comparison. **f**, V3 vs. V4 comparison.

Supplementary Figure 18 | Multidimensional scaling (MDS) embeddings of the in silico multivariate fMRI responses for controlling images found by applying multivariate RNC on high-level visual areas

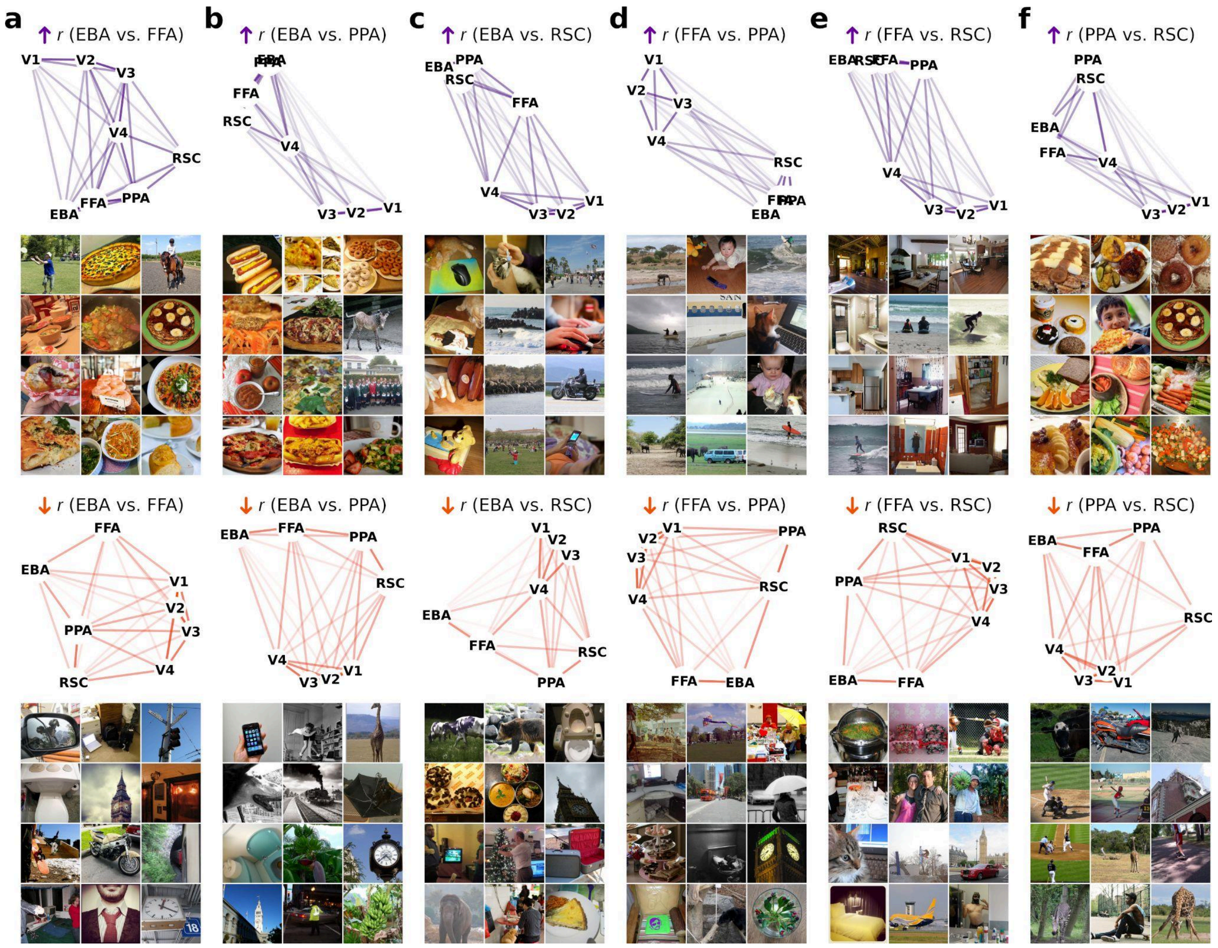

Twelve exemplar controlling images are shown for each control condition (all images come from the 73,000 NSD images). Each panel reflects MDS results and controlling images for a different pairwise comparison of areas. **a**, EBA vs. FFA comparison. **b**, EBA vs. PPA comparison. **c**, EBA vs. RSC comparison. **d**, FFA vs. PPA comparison. **e**, FFA vs. RSC comparison. **f**, PPA vs. RSC comparison.

Supplementary Table 1 | Early and mid-level visual areas' retained voxels for in silico fMRI responses from encoding models trained on NSD

	V1	V2	V3	V4
**Participant 1**	813 / 1,350 (60 %)	656 / 1,433 (46 %)	486 / 1,187 (41 %)	176 / 687 (26 %)
**Participant 2**	598 / 1,102 (54 %)	407 / 1,075 (38 %)	448 / 1,097 (41 %)	239 / 483 (49 %)
**Participant 3**	553 / 1,254 (44 %)	366 / 1,141 (32 %)	152 / 928 (16 %)	61 / 426 (14 %)
**Participant 4**	340 / 877 (39 %)	285 / 863 (33 %)	141 / 808 (17 %)	40 / 475 (8 %)
**Participant 5**	529 / 1,113 (48 %)	424 / 1,081 (39 %)	259 / 925 (28 %)	154 / 542 (28 %)
**Participant 6**	484 / 1,127 (43 %)	381 / 1,180 (32 %)	205 / 1,201 (17 %)	50 / 477 (10 %)
**Participant 7**	273 / 1,142 (24 %)	174 / 986 (18 %)	86 / 726 (12 %)	48 / 397 (12 %)
**Participant 8**	234 / 1,074 (22 %)	168 / 1,033 (16 %)	68 / 889 (7 %)	60 / 495 (12 %)

Each cell indicates the amount of retained voxels (i.e., voxels with noise ceiling signal-to-noise ratio (ncsnr) scores above 0.5) out of the total voxels, for a given participant and area.

Supplementary Table 2 | High-level visual areas' retained voxels for in silico fMRI responses from encoding models trained on NSD

	EBA	FFA	PPA	RSC
**Participant 1**	1,145 / 2,971 (39 %)	164 / 794 (21 %)	158 / 1,033 (15 %)	87 / 566 (15 %)
**Participant 2**	1,036 / 3,439 (30 %)	230 / 869 (26 %)	271 / 994 (27 %)	244 / 813 (30 %)
**Participant 3**	831 / 3,518 (24 %)	205 / 1,093 (19 %)	220 / 1,269 (17 %)	20 / 838 (2 %)
**Participant 4**	730 / 3,288 (22 %)	163 / 942 (17 %)	141 / 960 (15 %)	59 / 813 (7 %)
**Participant 5**	1,587 / 4,587 (35 %)	220 / 907 (24 %)	444 / 1,221 (36 %)	77 / 771 (10 %)
**Participant 6**	1,029 / 4,126 (25 %)	156 / 826 (19 %)	287 / 1,229 (23 %)	27 / 845 (3 %)
**Participant 7**	521 / 3,062 (17 %)	47 / 484 (10 %)	61 / 912 (7 %)	18 / 694 (3 %)
**Participant 8**	200 / 3,184 (6 %)	116 / 1,204 (10 %)	88 / 961 (9 %)	13 / 799 (2 %)

Each cell indicates the amount of retained voxels (i.e., voxels with noise ceiling signal-to-noise ratio (ncsnr) scores above 0.5) out of the total voxels, for a given participant and area.

Supplementary Table 3 | Early and mid-level visual areas' retained voxels for in silico fMRI responses from encoding models trained on the Visual Illusion Reconstruction (VIR) dataset

	V1	V2	V3	V4
**Participant 1**	419 / 2,444 (17 %)	444 / 3,018 (15 %)	326 / 2,556 (13 %)	20 / 899 (2 %)
**Participant 2**	427 / 2,850 (15 %)	343 / 2,927 (12 %)	257 / 3,120 (8 %)	65 / 1,089 (6 %)
**Participant 3**	232 / 2,045 (11 %)	185 / 2,536 (7 %)	130 / 2,763 (5 %)	16 / 1,916 (1 %)
**Participant 4**	80 / 2,637 (3 %)	38 / 2,921 (1 %)	17 / 2,336 (1 %)	12 / 1,552 (1 %)
**Participant 5**	144 / 2,210 (7 %)	74 / 2,032 (4 %)	53 / 2,222 (2 %)	40 / 1,971 (2 %)
**Participant 6**	608 / 2,614 (23 %)	640 / 2,909 (22 %)	588 / 3,023 (19 %)	139 / 1,338 (10 %)
**Participant 7**	609 / 1,892 (32 %)	701 / 2,113 (33 %)	635 / 1,930 (33 %)	241 / 986 (24 %)

Each cell indicates the amount of retained voxels (i.e., voxels with noise ceiling signal-to-noise ratio (ncsnr) scores above 0.5) out of the total voxels, for a given participant and area. Because area V4 of VIR participant 4 did not have voxels with ncsnr above 0.5, for this participant and area we instead lowered the ncsnr threshold to 0.4.

Supplementary Table 4 | In vivo fMRI retained voxels for the univariate RNC experiment

	V1	V4
**Participant 1**	35 / 382<br>(9 %)	25 / 323<br>(8 %)
**Participant 2**	225 / 487<br>(46 %)	173 / 383<br>(45 %)
**Participant 3**	283 / 647<br>(44 %)	125 / 284<br>(44 %)
**Participant 4**	211 / 425<br>(50 %)	96 / 289<br>(33 %)
**Participant 5**	157 / 389<br>(40 %)	66 / 286<br>(23 %)
**Participant 6**	262 / 428<br>(61 %)	167 / 375<br>(45 %)

Each cell indicates the amount of retained voxels (i.e., voxels with noise ceiling signal-to-noise ratio (ncsnr) scores above 0.4) out of the total voxels, for a given participant and area.

Supplementary Table 5 | In vivo fMRI retained voxels for the multivariate RNC experiment

	**V1**	**V4**
**Participant 1**	86 / 382<br>(23 %)	55 / 323<br>(17 %)
**Participant 2**	190 / 487<br>(39 %)	101 / 383<br>(26 %)
**Participant 3**	242 / 647<br>(37 %)	67 / 284<br>(24 %)
**Participant 4**	109 / 425<br>(26 %)	24 / 289<br>(8 %)
**Participant 5**	105 / 389<br>(27 %)	30 / 286<br>(10 %)
**Participant 6**	270 / 428<br>(63 %)	130 / 375<br>(35 %)

Each cell indicates the amount of retained voxels (i.e., voxels with noise ceiling signal-to-noise ratio (ncsnr) scores above 0.4) out of the total voxels, for a given participant and area.
